# Comparative transcriptome profiling of resistant and susceptible foxtail millet responses to *Sclerospora graminicola* infection

**DOI:** 10.1186/s12870-022-03963-5

**Published:** 2022-12-06

**Authors:** He Wang, Yanqing Han, Caijuan Wu, Baojun Zhang, Yaofei Zhao, Jiao Zhu, Yuanhuai Han, Jianming Wang

**Affiliations:** 1 College of Plant Protection, Shanxi Agricultural University, Taigu, 030801 Shanxi China; 2 College of Agriculture, Shanxi Agricultural University, Taigu, 030801 Shanxi China; 3 Shanxi Key Laboratory of Germplasm Innovation and Molecular Breeding of Minor Crop, Taiyuan, 030031 China

**Keywords:** Foxtail millet, Downy mildew, *Sclerospora graminicola*, Transcriptome, Differentially expressed genes, Resistance mechanism

## Abstract

**Background:**

Downy mildew of foxtail millet, which is caused by the biotrophic oomycete *Sclerospora graminicola* (Sacc.) Schroeter, is one of the most disruptive diseases. The foxtail millet-*S. graminicola* interaction is largely unexplored. Transcriptome sequencing technology can help to reveal the interaction mechanism between foxtail millet and its pathogens.

**Results:**

Transmission electron microscopy observations of leaves infected with *S. graminicola* showed that the structures of organelles in the host cells gradually became deformed and damaged, or even disappeared from the 3- to 7-leaf stages. However, organelles in the leaves of resistant variety were rarely damaged. Moreover, the activities of seven cell wall degrading enzymes in resistant and susceptible varieties were also quite different after pathogen induction and most of enzymes activities were significantly higher in the susceptible variety JG21 than in the resistant variety G1 at all stages. Subsequently, we compared the transcriptional profiles between the G1 and JG21 in response to *S. graminicola* infection at 3-, 5-, and 7-leaf stages using RNA-Seq technology. A total of 473 and 1433 differentially expressed genes (DEGs) were identified in the resistant and susceptible varieties, respectively. The pathway analysis of the DEGs showed that the highly enriched categories were related to glutathione metabolism, plant hormone signalling, phenylalanine metabolism, and cutin, suberin and wax biosynthesis. Some defence-related genes were also revealed in the DEGs, including leucine-rich protein kinase, Ser/Thr protein kinase, peroxidase, cell wall degrading enzymes, laccases and auxin response genes. Our results also confirmed the linkage of transcriptomic data with qRT-PCR data. In particular, LRR protein kinase encoded by Seita.8G131800, Ser/Thr protein kinase encoded by Seita.2G024900 and Seita. 2G024800, which have played an essential resistant role during the infection by *S. graminicola*.

**Conclusions:**

Transcriptome sequencing revealed that host resistance to *S. graminicola* was likely due to the activation of defence-related genes, such as leucine-rich protein kinase and Ser/Thr protein kinase. Our study identified pathways and genes that contribute to the understanding of the interaction between foxtail millet and *S. graminicola* at the transcriptomic level*.* The results will help us better understand the resistance mechanism of foxtail millet against *S. graminicola*.

**Supplementary Information:**

The online version contains supplementary material available at 10.1186/s12870-022-03963-5.

## Background

Foxtail millet is an important cereal crop in northern China with special characteristics such as tolerance to drought, barrenness and saline alkali soil [1]. This crop is environmentally friendly with low water and fertilizer consumption and is important for alleviating the current situation of water shortages in foxtail millet planting areas in northern China [2]. In addition, because of its small genome and self-pollination, foxtail millet is very suitable for use as a model crop for whole-genome research, biofuel research and photosynthesis research on C_4_ model crops [3].

In recent years, the occurrence of some plant diseases has increased due to global warming, changes in cultivation conditions, and the popularization of single and homogeneous high-yield and high-quality varieties in large areas. Downy mildew of foxtail millet caused by *Sclerospora graminicola* (Sacc.) has recently emerged as the most destructive disease, restricting the production of foxtail millet in China, Japan, and Russia [4, 5]. Previous research has shown that when foxtail millet plants are infected by *S. graminicola*, yields decrease by 5–10% and can even be reduced by more than 50% in years when the infection is serious [6]. *S. graminicola* is an obligate biotrophic oomycete with a genome size of approximately 360 Mbps [5], which is larger than those of the oomycete pathogens *Plasmopara halstedii*, *Hyaloperonospora arabidopsidis*, *Phytophthora infestans* and *Phytophthora sojae* [5, 7]. Interestingly, *S. graminicola* infects and causes different symptoms throughout the life cycle of foxtail millet, including the symptoms ‘bud death’, ‘greyback’, ‘white tip’, ‘white hair’, and ‘green ear’ or ‘hedgehog panicle’. Several fungicides were shown to be effective against *S. graminicola* based on seed treatment, but the pathogen has become resistant to fungicides, which also causes environmental pollution [8]. Plant breeding for disease resistance has become the most attractive way to control this disease effectively and is an environmentally friendly method for disease management. The identification and exploitation of disease resistance genes in germplasm are the central focus in foxtail millet breeding programs.

Plant growth and development processes are mainly controlled by phytohormones [9, 10], which also play key roles in regulating immune responses to pathogen invasions [11, 12]. Ethylene (ET), jasmonic acid (JA) and salicylic acid (SA) play a central role in the regulation of plant immunity [4, 13]. In addition, other plant hormones such as auxins, abscisic acid (ABA), cytokinins, gibberellins, and brassinosteroids have also as key regulators of plant immunity [14, 15]. Indole-3-acetic acid (IAA) negatively regulates the resistance of rice to pathogen infection [16]. Treatment of rice with IAA or 2,4-Dichlorophenoxyacetic acid (2,4-D) can stimulate the proliferation of the pathogen *Xanthomonas oryzae* pv. *oryzae* and makes rice more susceptible to pathogens [17]. Moreover, *Xoo* infection can induce the accumulation of IAA in the host [17]. The concentration of ABA in tobacco increases with the infection of tobacco mosaic virus (TMV), and the external application of ABA can enhance resistance to TMV [18]. Brassinolide (BR) treatment can alleviate the symptoms of rice blast and bacterial blight [19]. Exogenous GA could increase the resistance of rice to *Magnaporthe oryzae* [20].

Pathogen infection can induce a host defence response and changes in carbohydrate and starch metabolism. The pathogen withdraws carbohydrates from the plant and attempts to manipulate the plant to use carbohydrates for their development. Moreover, host plants can resist the infection of germs by recombining carbon flux [21]. It was found that after *Botrytis cinerea* infected tomato leaves, the expression of genes involved in carbohydrate increased significantly, and the sugar content also changed with the change in infection time but had little effect on the starch content [21]. By measuring the starch content of citrus plants infected by the Huanglongbing (HLB) pathogen, it was found that the starch content in infected citrus leaves was significantly higher than that in healthy leaves [22]. The contents of soluble sugar and starch in *Citrus sinensis* leaves are greatly affected by HLB infection, which increased with the extension of infection time and decreased slightly in the later stage, indicating that HLB infection affected the synthesis and transportation of photosynthetic products and promoted their accumulation [23].

High-throughput RNA sequencing has gained popularity for exploring gene expression changes in plants during oomycete pathogen infections [24, 25]. This technology provides insight into expression changes during the basic defence response and helps to elucidate the complex resistance mechanism in plants by comparing the gene expression between susceptible and resistant varieties after infection. Transcriptome analysis and gene expression studies have been performed to evaluate interactions between oomycete pathogens in plants. Kulkarni et al. (2016) compared transcript levels between resistant and susceptible pearl millet varieties to *S. graminicola*, and the R genes, PR proteins, HR-induced proteins, and transcripts for the skp1 protein, purothionin, V-type proton ATPase, and genes involved in plant hormonal signalling transduction were determined to potentially be involved in disease defence [26]. Comparative transcriptome analysis demonstrated that several protease inhibitors, chitinases, defensin, PR-1, and a downy mildew susceptibility factor respond to *Phytophthora parasitica* infection in tomato [27]. Transcriptome analysis of *S. graminicola*-infected foxtail millet found that plant hormones, such as gibberellin, jasmonic acid and abscisic acid, play an important regulatory role in the formation of hedgehog panicles [4]. In *Solanum tuberosum* and tobacco, pathogenesis-related proteins were found to be induced in resistant genotypes after *Phytophthora infestans* and *Phytophthora capsici* infection [28, 29].

In this study, we performed a comparative analysis between resistant and highly susceptible foxtail millet varieties to reveal the plant resistance mechanisms at the phenotypic, physiological and transcriptomic levels. We examined the microstructure, enzyme activity and gene differential expression patterns of these two foxtail millet varieties in response to *S. graminicola* infection to facilitate the understanding of the transcriptional regulation pattern of resistant and susceptible foxtail millet varieties and promote further research for molecular breeding.

## Results

### Identification and characterization of two foxtail millet varieties with different responses to *S. graminicola* infection

Five foxtail millet varieties were evaluated for resistance to downy mildew after inoculation by mixing oospores and seeds in the field and greenhouse. The JG21 variety had the highest susceptibility (disease grade 9), its reputation having the least resistance to the pathogen, with a "grey back" incidence of downy mildew of 52.1% and 60.7% under field and greenhouse conditions, respectively (Table S1 and Fig. S1). The "grey back" incidence rate of the JG40 variety was slightly lower than that of JG21, with values of 47.6% and 54.4% under field and greenhouse conditions, respectively. The incidence rates of FX3 and JG42 were lower than those of JG21 and JG40. G1 exhibited high resistance (disease grade 1) to *S. graminicola*, with a "grey back" incidence of 0.00% under both field and greenhouse conditions (Table S1 and Fig. S1). According to the above result, we selected highly susceptible JG21 and highly resistant G1 varieties for subsequent RNA-Seq analysis.

### Phenotypic symptoms and transmission electron microscopy observations

**Fig. 1:**
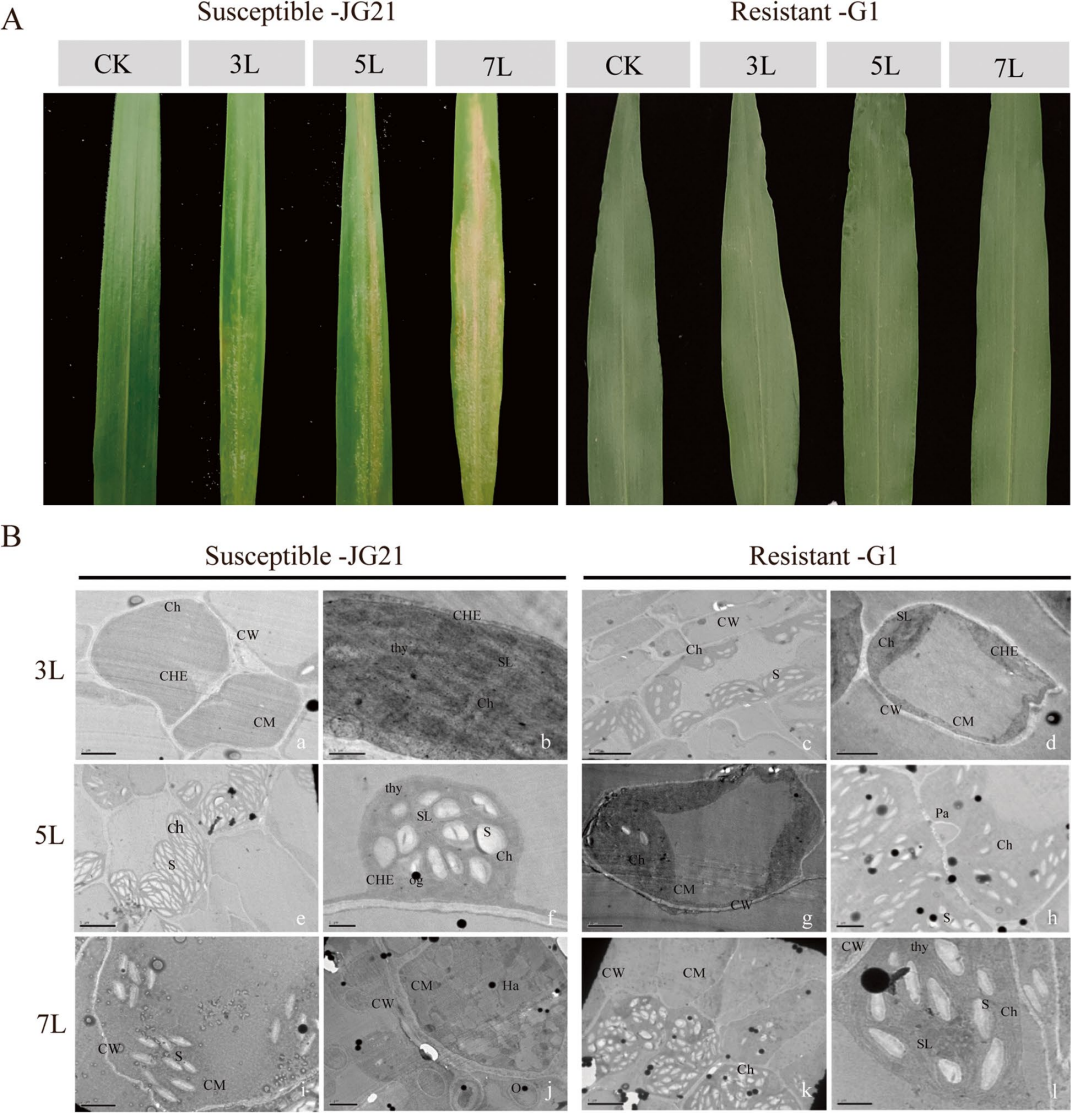
Phenotypic symptoms and ultrastructural changes of resistant and susceptible varieties at different infection stages. Abbreviations in the figure: Ch, chloroplast; thy, thylakoid; SL, stroma lamella; CW, cell wall; CM, cell membrane; Ha, haustorium; S, starch granules; CHE, chloroplast membrane; og, osmiophilic granule; Pa, papilla; O, oospores

The symptom of "grey back" mildew appeared at the seedling stage after *S. graminicola* infection in the JG21 variety. At the 3-leaf stage (3L), a sparse grey-white mould layer appeared on the backs of leaves, and then the white mould layer became denser at the 5-leaf stage (5L), resulting in a large number of sporangiophores and sporangia. At the 7-leaf stage (7L), apart from a large number of mould layers and asexual spores, the leaf colour gradually from green to yellow-white (Fig. 1A). However, the leaves of the resistant variety G1 did not show obvious symptoms after infection with *S. graminicola* (Fig. 1A). To clarify the ultrastructural changes in cell morphology in resistant and susceptible varieties, the leaf tissues of resistant and susceptible varieties at 3L, 5L and 7L after inoculation were observed by TEM (Fig. 1B). At 3L, the mesophyll cell and chloroplast double membrane of the susceptible variety JG21 were intact, and there were no markedly abnormal findings. The chloroplast granular layer was clear, and there were fewer starch grains (Fig. 1B-a, b). The mesophyll cells of G1 were arranged in a rectangular shape, and the cell membrane and cell wall were intact and clear; the chloroplasts were spindle-shaped and numerous; the granular layered structure was clear; and starch granule accumulation was observed in mesophyll cells (Fig. 1B-c, d**).** At 5L, the leaf ultrastructure of the susceptible variety JG21 changed greatly. The most typical symptom was that the chloroplast was obviously deformed from a spindle to an oval or spherical shape. The number of white and spindle-shaped starch grains increased significantly, covering almost all chloroplast structures (Fig. 1B-e). The internal structure of the chloroplast was damaged, the thylakoids were loosely arranged, the granular layered structure was disordered, the arrangement was irregular, and the number of osmium-philic granules gradually increased (Fig. 1B-f). The cell walls of the resistant variety were relatively complete; the chloroplast granular layer was clear (Fig. 1B-g); there were more starch grains in the chloroplast; and there were papillary protrusions on the inner side of the cell wall (Fig. 1B-h). At 7L, pathogen infection caused severe damage to the chloroplast double-layer membrane of the susceptible variety JG21. Both the membrane structure and the granular layered structure were degraded, and the thylakoid membrane was completely destroyed. The inner and outer chloroplast membranes were obviously degraded and disappeared, the chloroplast was completely disintegrated, and starch grains with gelatinized edges were scattered in the cytoplasmic matrix (Fig. 1B-i). The formation of haustoria and oospores was observed in severely damaged cells (Fig. 1B-j). In the resistant variety G1, the chloroplasts in the cells were partially deformed, and some of the cells became vacuoles without an organellar structure (Fig. 1B-k). In addition, the chloroplast basal lamellae were disordered (Fig. 1B-l).

### Physiological changes in the two foxtail millet varieties in response to *S. graminicola* infection

**Fig. 2:**
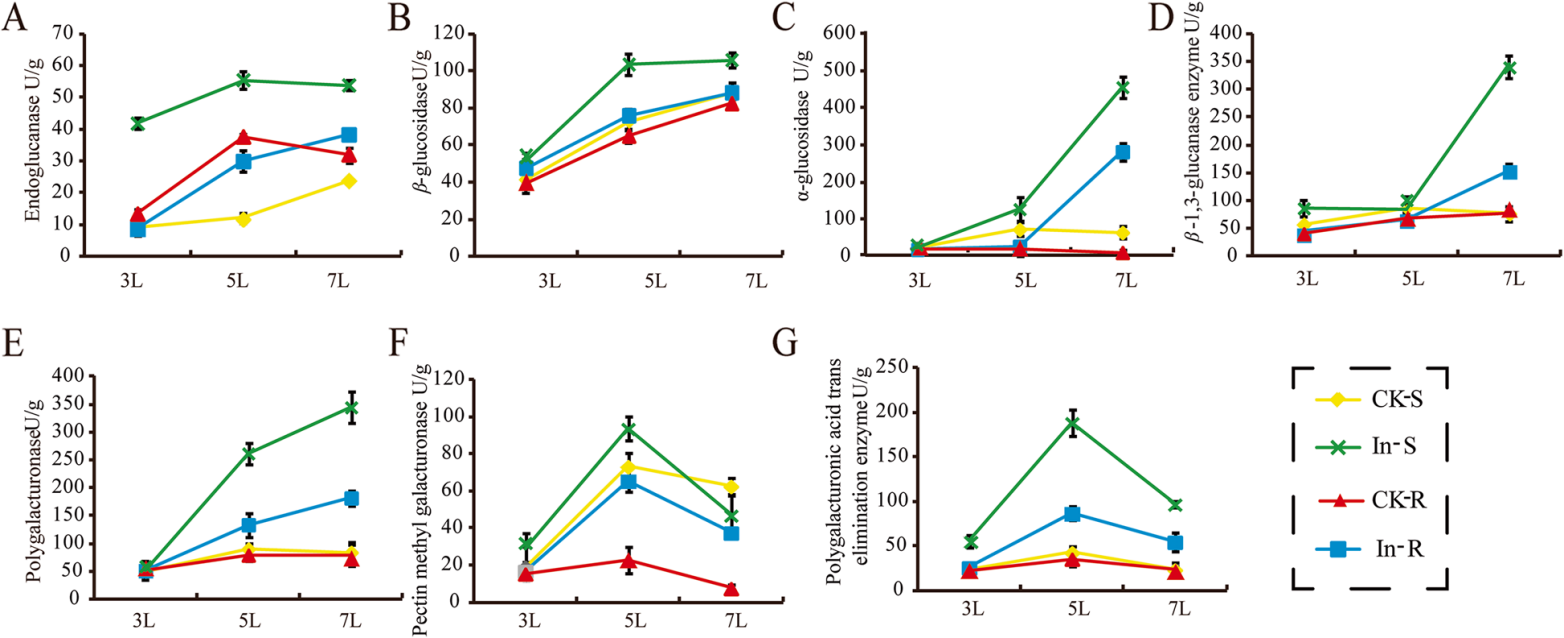
Changes in cellulases, hemicellulases and pectinases in foxtail millet leaves infected by *S. graminicola*. CK-S: uninoculated JG21, treated-S: inoculated JG21, CK-R: uninoculated G1, treated-R: inoculated G1

During their interaction with the host, pathogens can secrete a variety of cell wall degrading enzymes (CWDE), which can destroy the cell wall of the host, lead to necrosis symptoms or accelerate the development of the disease course, and provide nutrients such as sugar sources for the growth and reproduction of pathogenic bacteria [30]. To test this possibility in this study, we measured the activities of seven CWDE, including Cx, *β*-G, α-glucanase, *β*-1,3-glucanase polygalacturonase, PMG and PGTE, which act as virulence factors and elicitors of the host defence response. We determined the activities of the CWDE at 3L, 5L and 7L after infection with *S. graminicola* (Fig. 2). Expectedly, CX and PGTE activity was significantly higher in the susceptible variety JG21 than in the resistant variety G1 in all infection periods (Fig. 2A, D, F, G). Moreover, the activities of *β*-G, α-glucanase and PMG were significantly higher in JG21 than in G1 at 5L and 7L, with no considerable differences at 3L between the JG21 and G1 varieties after *S. graminicola* infection (Fig. 2B, C, E). The activities of cellulose-, hemicellulose- and pectin-degrading enzymes showed obvious differences in both varieties, and higher activities were found in the susceptible variety JG21. It can be speculated that the CWDE likely to degraded the host cell wall to obtain enough nutrition for the production of zoosporangia at the important of “grey back” stage at 5L.

### Transcriptomic response of the resistant and susceptible varieties under *S. graminicola* infection

**Fig. 3:**
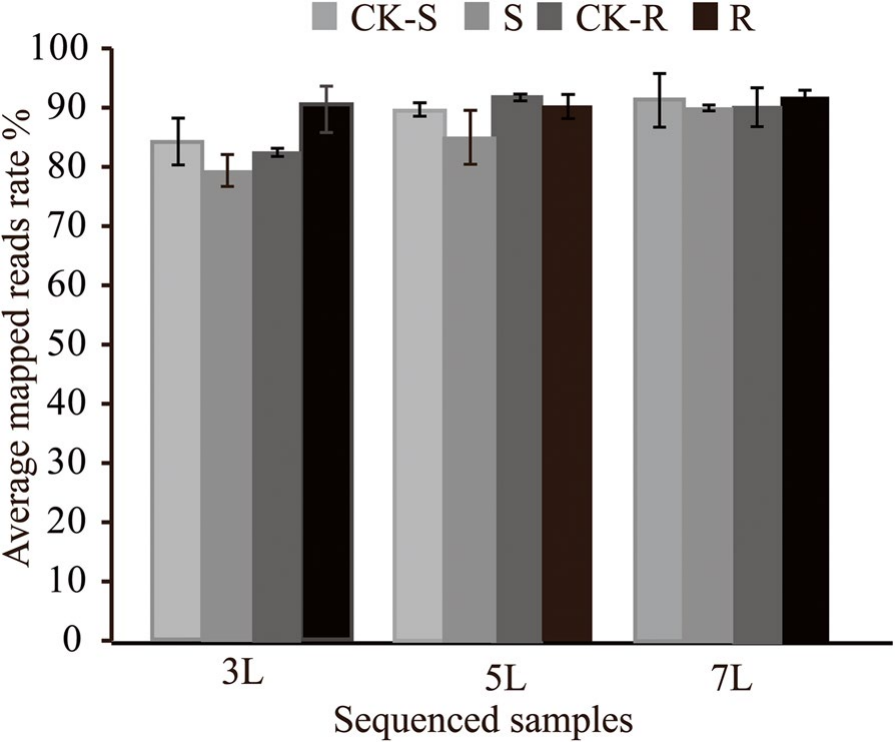
Reads mapped against the reference genome in each sample. Note: Each column represents the average mapped read ratio of three biological replicates

To clarify the molecular mechanism of the interaction between foxtail millet and *S. graminicola*, a total of 36 RNA samples isolated at 3L, 5L and 7L from G1 and JG21 leaves infected with *S. graminicola* were subjected to RNA-Seq. A total of 328.19 Gb of clean data was obtained after removing low-quality reads. The percentage of Q30 bases was 95.23%, and the average GC percentage was 57.67% (Table S2). After low-quality regions and adapter sequences were removed, an average of 30,498,412.80 bp clean reads were obtained and mapped to the reference genome with an average mapping ratio of 86.70% (Fig. 3). The results showed that the sequence quality of the transcriptome was sufficient for further analysis.

**Fig. 4:**
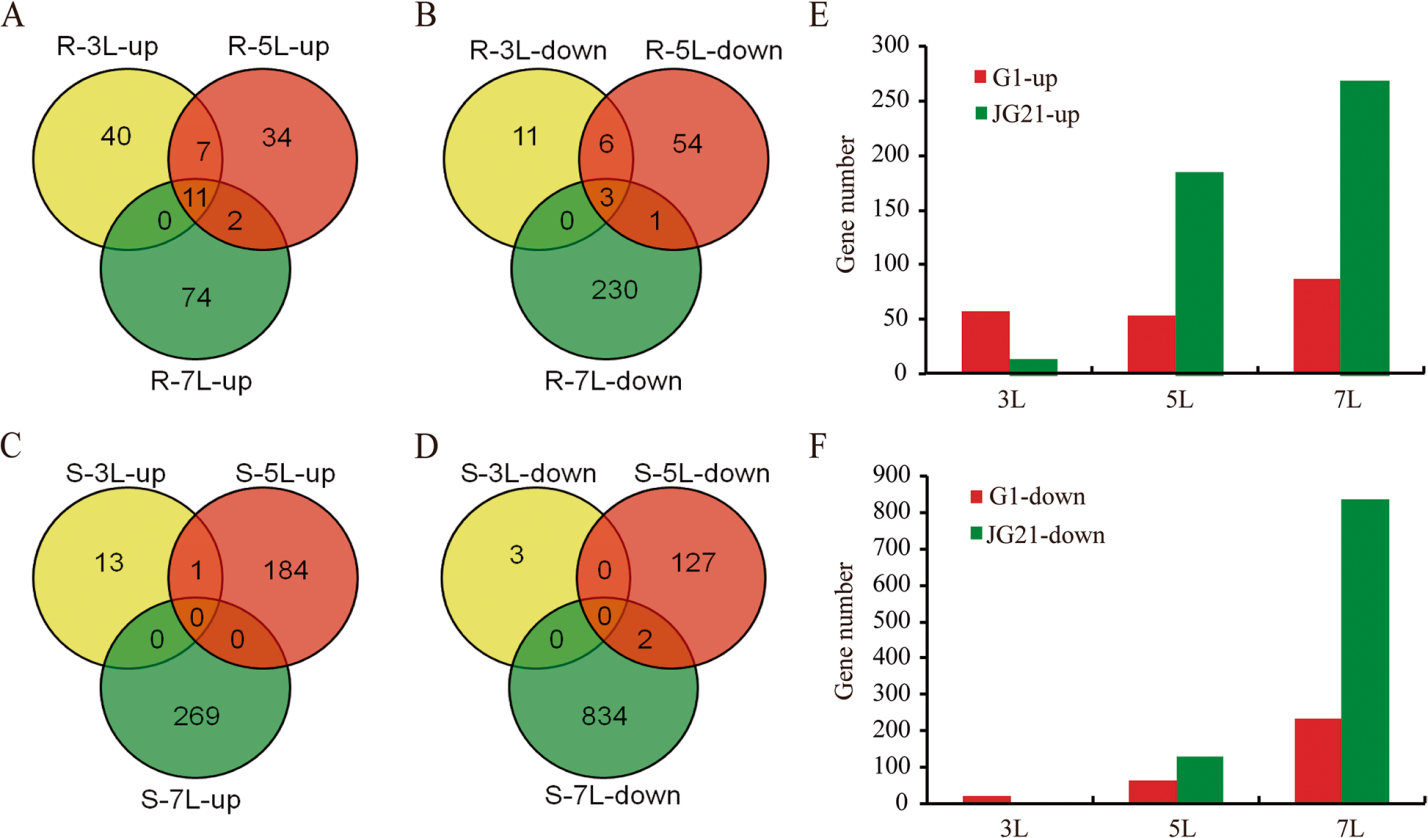
Overview of global changes in differentially expressed genes across different periods in *S. graminicola*-infected foxtail millet varieties G1 and JG21. Venn diagrams of susceptible (**A**) up-regulated and (**B**) down-regulated and resistant (**C**) up-regulated and (**D**) down-regulated genes detected in infected samples relative to controls. **E** Heatmap analysis of differentially expressed genes from susceptible JG21 and resistant G1 across stages. Numbers within regions in the Venn diagram indicate common and unique genes within each sector

Global transcriptional responses of foxtail millet varieties resistant and susceptible to *S. graminicola* infection from 3 to 7L were investigated using RNA-Seq. DEGs were identified for *S. graminicola-*infected varieties relative to control using an FDR ≤ 0.01 and a log fold change ≥ 1.0. A Venn diagram comparing DEGs in inoculated versus uninoculated foxtail millet at the three time points revealed 78, 118, and 321 DEGs in the resistant variety at 3L, 5L and 7L, respectively, among which 51, 88, and 304 DEGs were unique to each time point (Fig. 4A, B, E). In the susceptible variety, 17, 314 and 1105 DEGs were identified at L3, L5 and L7, respectively, among which 16, 311 and 1103 DEGs were unique to each time point (Fig. 4C, D, F). After being infected by the pathogen, the numbers of up-regulated and down-regulated DEGs increased in both varieties (Fig. 4E, F). We speculated that an increasing number of DEGs were involved in the interaction between foxtail millet and *S. graminicola*.

In the susceptible variety, the maximum number of DEGs (1211) occurred at 5L relative to the uninfected controls. Smaller numbers of DEGs (1104) were observed at 7L, and the minimum number of DEGs (8) was observed at 3L (Fig. 4C, D). In addition, as shown in Fig. 4 (Fig. 4A, B, C, D), it can be seen that the DEGs were not coexpressed in JG21(0-up, 0-down) or that there was a relatively small number of coexpressed genes (11-up, 3-down) in G1. We speculated that the number genes involved in disease regulation and were different at different stages when the pathogen infected the host.

### Common Gene Ontology enrichment responses to *S. graminicola* infection between JG21 and G1

**Fig. 5:**
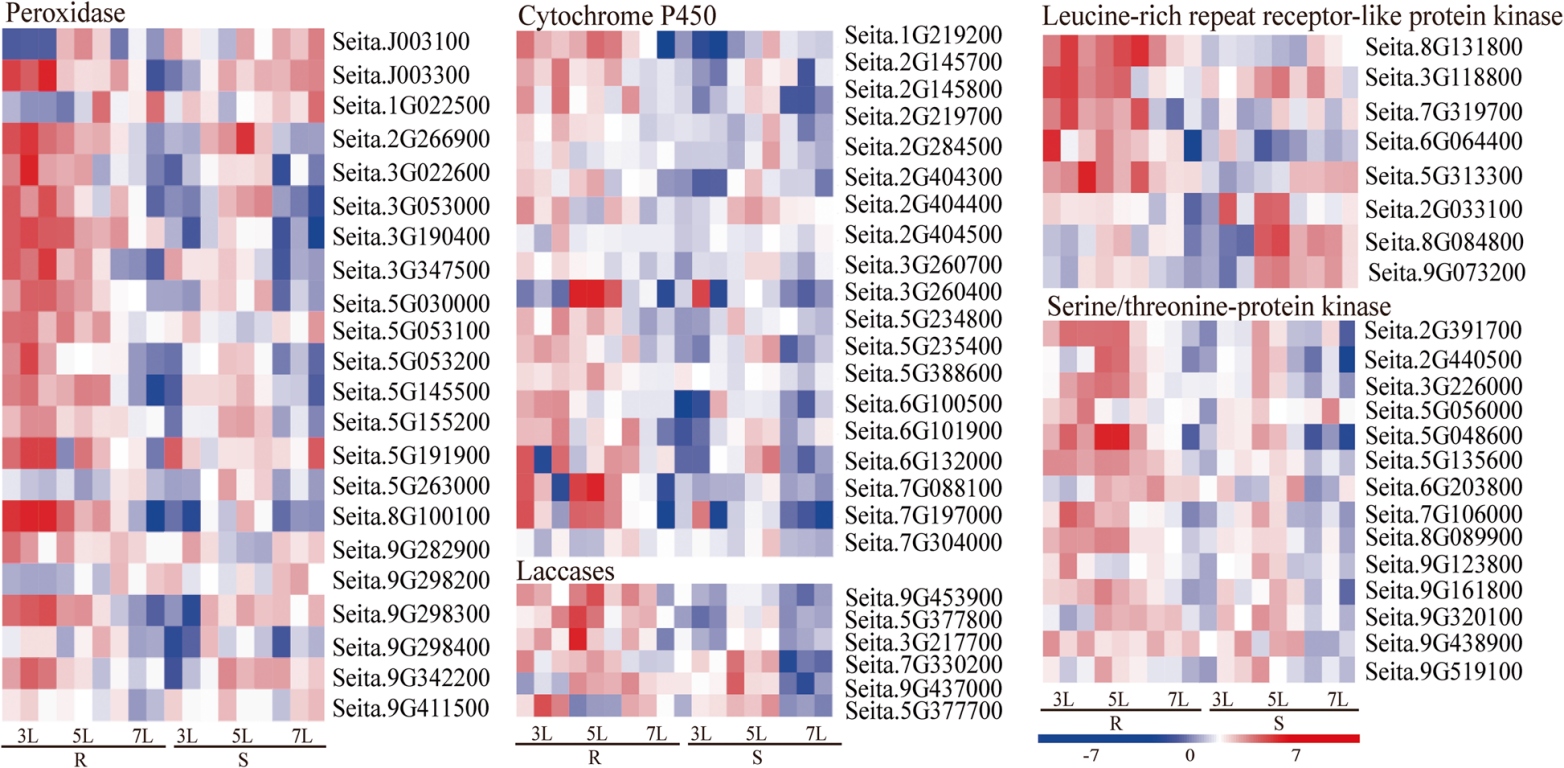
Differentially expressed genes associated with peroxidase, cytochrome P450, laccases, leucine-rich repeat receptor-like protein kinase genes, and serine/threonine-protein kinase in *S. graminicola*-infected susceptible JG21and resistant G1 varieties relative to the control at 3L, 5L, and 7 L

To explore the molecular functions that were responsive to *S. graminicola* infection, we performed Gene Ontology term enrichment analysis of the DEGs. All GO terms were assigned to three groups: biological process (BP), cellular component (CC) and molecular function (MF). In the BP category protein phosphorylation (GO:0,006,468) is one of the critical processes in plant‒pathogen interactions that can regulate signal transduction in cells. For example, MAPK is activated, and the expression of defence genes can be induced by the phosphorylation cascade, thus improving plant resistance. The MF terms related to serine/threonine kinase activity (GO: 000,674), peroxidase activity (GO: 0,004,601), haem-binding (GO: 0,020,037) and laccases (GO: 0,048,046) were important functional groups involved in secondary metabolite production and important defence pathways. Up-regulated and down-regulated DEGs were associated with the plant defence response. Examples of peroxidase genes, cytochrome P450, laccases, leucine-rich-repeat receptor-like kinases (LRR-LRKs) and serine/threonine-protein kinases (STKs) are shown in Fig. 5. Sixty-eight percent of peroxidase genes were dramatically increased in G1 after *S. graminicola* infection at the early stage. Similar to peroxidase genes, 84% of the differentially expressed cytochrome P450 genes were up-regulated in G1, and 16% of them were decreased at the same stage. The differentially expressed LRR-LRKs (62%) and STKs (42%) were up-regulated at 3L and 5L in G1. In addition, three of six laccases (50%) had a much higher expression level in G1 than in JG21 at all infection stages. Overall, pathogen infection induced global gene expression changes in both varieties.

### Identification of DEGs in commonly enriched KEGG pathways in both varieties

**Fig. 6:**
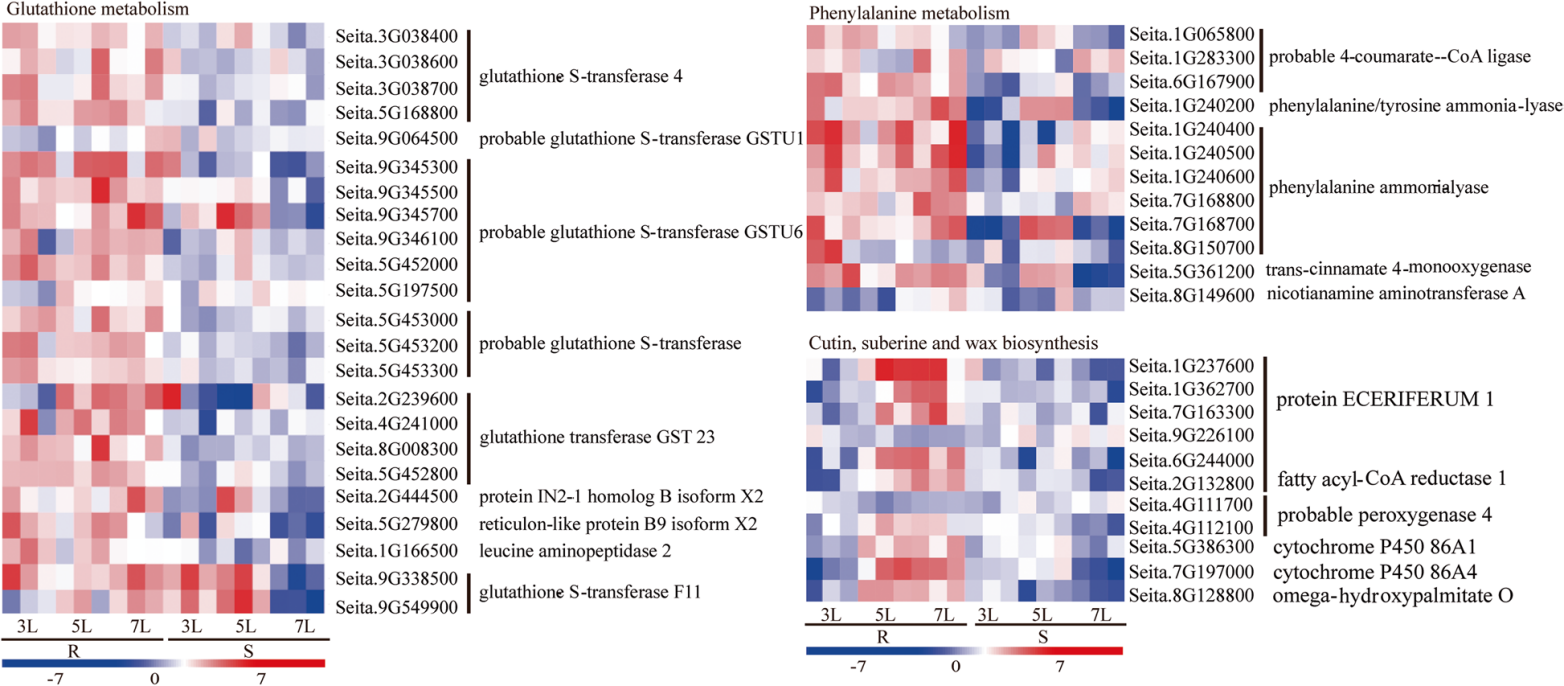
Differentially expressed genes associated with phenylalanine metabolism, glutathione metabolism, cutin and suberin and wax biosynthesis in susceptible JG21and resistant G1 foxtail millet varieties after *S. graminicola* infection relative to controls at 3L, 5L, and 7L

To identify important genes that might be involved in metabolism or biosynthesis critical for downy mildew resistance of foxtail millet in G1, pathway enrichment analyses were performed using KEGG. Only a few defence-associated biosynthetic pathways involving cutin, suberin or wax biosynthesis were enriched in the transcriptome of the resistant varieties, while more pathways, such as glutathione metabolism andphenylalanine metabolites, were significantly enriched in both resistant and susceptible varieties after inoculation. Comparisons of the expression levels of DEGs in these enriched pathways revealed that most genes were up-regulated in the resistant variety but down-regulated in the susceptible JG21. Among them, glutathione S-transferase genes were greatly up-regulated after *S. graminicola* inoculation in G1 (Fig. 6). In contrast, the differentially regulated glutathione S-transferase genes exhibited distinctive expression patterns in the susceptible variety.

Phenylalanine ammonia-lyases (PALs) are involved in the synthesis of both lignin and phytoalexins to inhibit pathogens from penetrating cell walls [31]. Six PAL genes (Seita.1G240200, Seita.1G240400, Seita.1G240500, Seita.7G240600, Seita.7G168700 and Seita.7G168800) involved in the phenylalanine metabolism pathways were up-regulated in G1 and down-regulated in JG21 (Fig. 6). Genes encoding 4-coumarate-CoA ligase (Seita.1G065800, Seita.1G283300 and Seita.6G167900) and trans-cinnamate 4-monooxygenase (Seita.5G361200) were up-regulated and involved in lignin biosynthesis through the phenylpropanoid biosynthesis pathway. The defence-related gene *PAL* was also significantly up-regulated in G1, which showed opposite expression patterns in JG21 after *S. graminicola* induction.

Pathway enrichment analysis revealed that the cutin, suberin and wax biosynthesis pathways were markedly enriched by DEGs. Overall, 11 of the DEGs were significantly differentially expressed between the resistant and susceptible varieties (Fig. 6). All DEGs were down-regulated at 3L in both resistant and susceptible varieties. With further pathogen infection, approximately 81.8% (9) of the DEGs were rapidly and strongly induced at 5L and 7L in the resistant variety G1. Three CER1 genes (Seita.1G237600, Seita.1G362700 and Seita.7G163300) were also dramatically induced and were involved in cutin and wax synthesis. In particular, the Seita.1G237600 gene showed 274.1- and 119.4-fold expression increases. Cytochrome 450 86A1/86A4 (Seita.7G197000), as the key enzyme of suberin synthesis, was found to be greatly up-regulated, with 45.2- and 70.0-fold expression at 5L and 7L, respectively, in the resistant G1 variety. In contrast, these genes were generally suppressed or not significantly regulated at all infection stages in JG21 (Fig. 6). Divergent patterns of expression in the resistant and susceptible varieties suggest that these genes might be essential for foxtail millet resistance to *S. graminicola* in the G1 variety.

### Genes related to hormonal pathways in response to downy mildew challenge

**Fig. 7:**
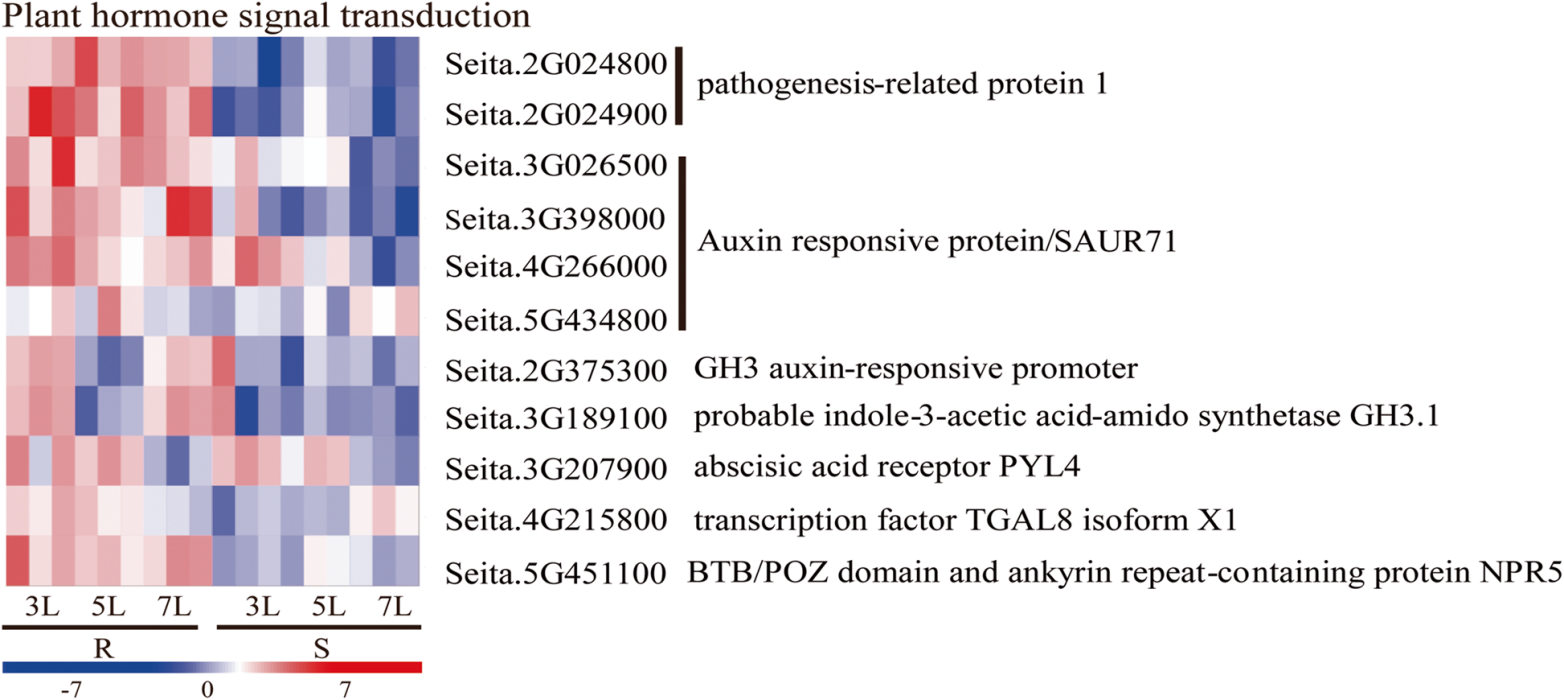
Differentially expressed genes associated with plant defences and plant hormone signal transduction pathways in *S. graminicola-*infected susceptible JG21 and resistant G1 foxtail millet varieties relative to controls at 3, 5, and 7L

Phytohormones such as SA, JA, and ET are known to play major roles in regulating plant defence responses against various pathogens [32]. A total of 11 DEGs were identified as being involved in hormone signal transduction pathways, such as those involving auxin (IAA), salicylic acid (SA), jasmonic acid (JA), abscisic acid (ABA) and gibberellic acid (GA), all of which play an important role in downy mildew resistance (Fig. 7). Five genes (3 SAUR71, 1 GH3 auxin-responsive promoter and 1 GH3.1) were up-regulated and significantly induced in the resistant variety G1, except Seita.5G434800, which was also down-regulated in JG21. PR1 is the marker gene of SA and JA signal transduction. In this study, two PR-1 genes, Seita.2G124900 and Seita.2G124800, were significantly induced and up-regulated in G1 but down-regulated in JG21. Moreover, DEGs of TGAL8 and NPR5 showed opposite expression trends in the resistant G1 and susceptible JG21, respectively (Fig. 7). In addition, PYL4 (Seita.3G207900), which is associated with ABA metabolism, was not significantly differentially expressed in either variety (Fig. 7). Our finding that defence-related genes, including PR and auxin-responsive genes, showed opposite expression patterns between G1 and JG21 after *S. graminicola* infection indicates that these genes play essential roles in downy mildew resistance in G1.

### Identification of DEGs in enriched in starch and sucrose metabolism and the fatty acid elongation pathway in the susceptible variety JG21

**Fig. 8:**
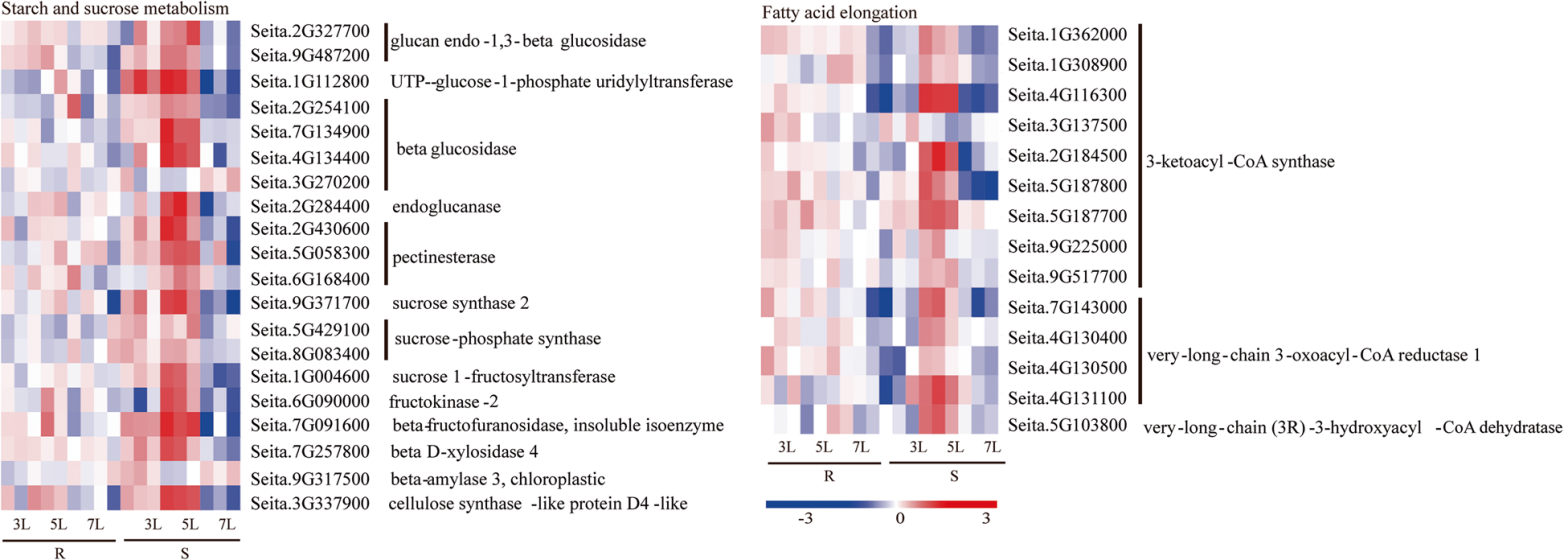
Differentially expressed genes associated with starch and sucrose metabolism in *S. graminicola-*infected susceptible JG21 and resistant G1 foxtail millet varieties relative to controls at 3L, 5L, and 7L

Plant pathogen infection leads to the deposition of starch molecules in the chloroplast and the accumulation of sucrose in infected plant cells [33, 34]. We investigated the expression pattern of transcripts related to starch and sucrose metabolism under *S. graminicola* infection (Fig. 8). The expression of most genes related to sugar metabolism was also enhanced in the JG21 leaves (Fig. 8). Notably, beta-amylase (*BMY*) and sucrose-catabolism genes (*SUSY*) showed the highest expression levels at 5L following *S. graminicola* attack in JG21, and these genes can promote the formation of glucose and fructose. Moreover, the expression of sucrose phosphate synthase (*SPS*), *β*-fructofuranosidase, which plays key roles in the sucrose synthesis process, also only markedly increased and was up-regulated at 5L in JG21. Consistently, seven enzymes, glucan endo-1,3-*β*-glucosidase, β-glucosidase (β-G), pectinesterase (PE), sucrose 1-fructosyltransferase (1-SST), fructokinase-2 (FRK), UTP-glucose-1-phosphate uridylyltransferase and beta-D-xylosidase 4, involved in cellulose hydrolysis and sucrose accumulation were up-regulated at 5L in JG21, which was the opposite expression pattern reported in G1 in response to *S. graminicola* infection. We speculate that the pathogen hijacked plant sucrose transporters during its attach, increased sugar outflow from infected sites, and eventually led to pathogen growth and plant defence at 5L.

The primary metabolism included the elongation of fatty acids. Genes associated with fatty acid elongation, including nine 3-ketoacyl-CoA synthases, 4 very-long-chain 3-oxoacyl-CoA reductases and 1 very-long-chain (3R)-3-hydroxy acyl-CoA dehydratase, were also generally up-regulated at 5L in the susceptible variety. The gene expression level of these DEGs was lower than that at 5L in G1 infected by *S. graminicola* (Fig. 8). In contrast, the expression of the DEGs was slightly induced and up-regulated at 3L and then gradually decreased until 7L in the resistant variety (Fig. 8). Fatty acid elongation-related genes were suppressed by the pathogen in the G1 accession but somehow induced in the JG21 variety, consistent with the concept that *S. graminicola* is likely to be a fatty acid auxotroph that requires lipids from the host.

### Validation of transcriptomics data by RT‒qPCR

**Fig. 9:**
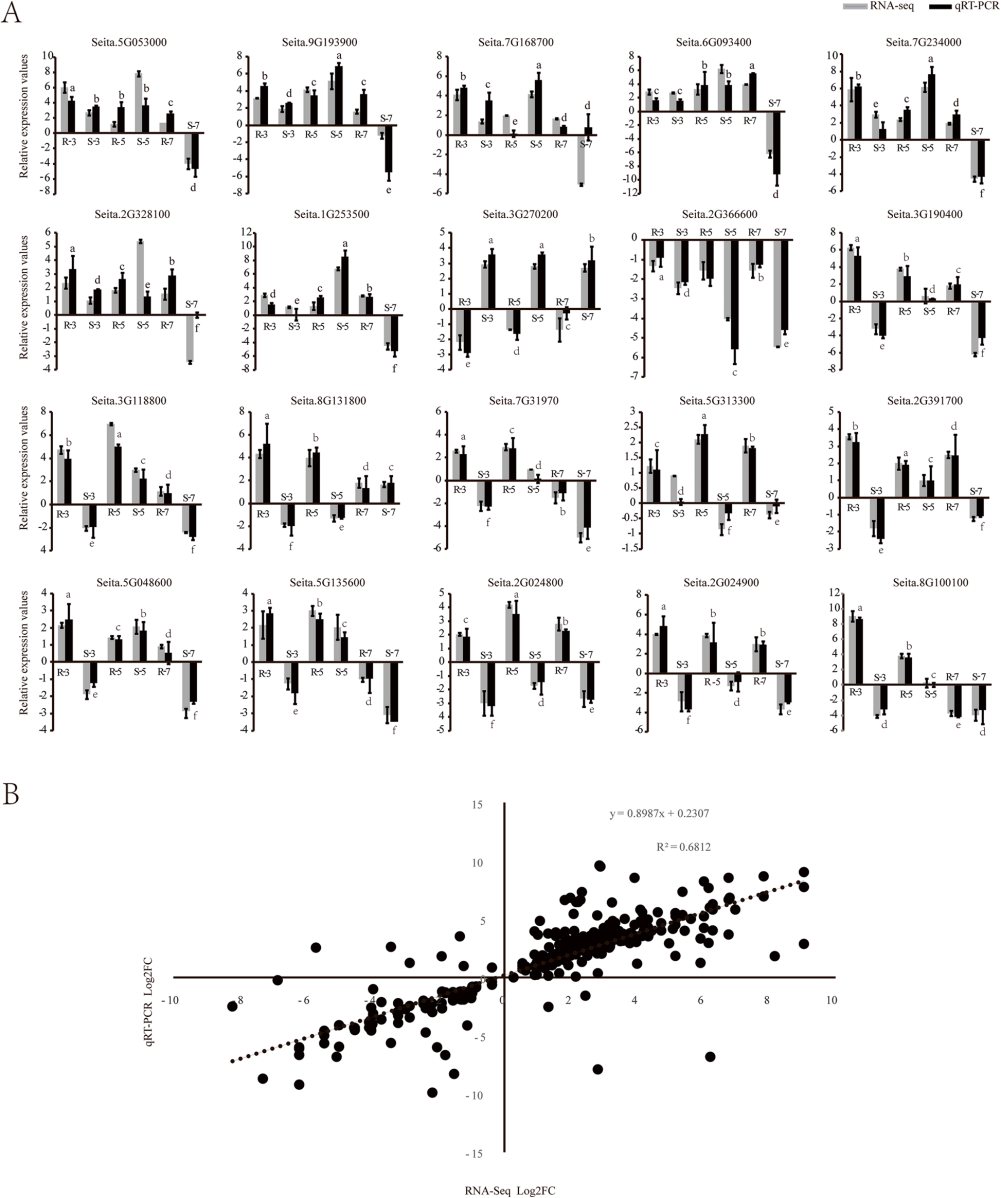
Expression levels of the DEGs from 3L, 5L, and 7L post inoculated JG21 and G1 foxtail millet were analysed. The log_2_(fold change) of the transcript levels in the inoculated samples compared to those in mock inoculated foxtail millet is shown. The error bars represent standard errors for three replicates of qRT‒PCR assays. The lowercase letters above the histogram indicated the statistical significance (Duncan’s multiple range test) at the level of 0.05 (*p* < 0.05). B. Correlation of transcript expression between RNA-Seq and qRT‒PCR results

To verify the differentially expressed genes based on RNA-Seq data, twenty DEGs potentially involved in the defence against *S. graminicola* and representing different expression patterns in both the resistant and susceptible varieties were selected for qRT‒PCR analysis. The relative expression values of all the tested genes were calculated using the constitutively expressed Actin gene. The results also showed a linear correlation between RNA-Seq and qRT‒PCR expression values based on R^2^(correlation coefficient of 0.68). The expression profiles of all tested genes resulting from qRT‒PCR were in line with our RNA-Seq data analysis results (Fig. 9).

## Discussion

Downy mildew is a serious threat to foxtail millet cultivation in China, especially to some elite varieties, such as JG21, that are highly susceptible to this pathogen. Although some varieties have been identified to have resistance to downy mildew, they are often poor performers in terms of agronomic traits. Improving host resistance to downy mildew and breeding resistant varieties would markedly benefit foxtail millet growers, but little is known about the resistance mechanism. Here, RNA-Seq technology was employed for the first time to investigate the resistance mechanisms in the early responses of resistant and susceptible foxtail millet to *S. graminicola* infection. Sixty-nine genes were enriched in GO terms such as the response to protein phosphorylation, serine/threonine kinase activity, and peroxidase activity (Fig. 4). By KEGG metabolic pathway analysis, phenylalanine metabolism, glutathione metabolism, cutin, suberin and wax biosynthesis, and plant hormone signal transduction were found to be significantly enriched in the resistant variety G1 (Figs. 5 and 6). These enriched DEGs and important pathways may play prominent roles in the regulation of resistance to *S. graminicola* in foxtail millet.

Through transcriptome analysis, we found some important genes involved in the regulation of disease resistance. The plant R gene plays an extremely important role in plant disease resistance. Plant R gene products can recognize the corresponding Avr-derived signals, activate downstream signal transduction and trigger complex defence reactions [35]. In this study, it was found that 8 leucine-rich repeats of receptor-like protein kinases (LRR-RLKs) and serine/threonine kinases (STKs) were significantly enriched in GO terms and showed similar protein kinase activity in resistant and susceptible varieties. Eight LRR-RLKs were observed to be regulated during the interaction between foxtail millet and *S. graminicola.* This suggests that these types of genes play key roles in the resistant G1 early response to downy mildew infections [36]. LRR-RLKs, through their extracellular LRR domain, can trigger immune signalling to promote plant resistance against pathogens [37–39]. For qRT-PCR verification, the leucine-rich repeat receptor-like protein encoded by Seita.6G181300 was up-regulated by 20.18-fold, 15.68-fold and 3.46-fold from 3 to 7L stage in resistant variety G1, respectively. However, the gene was down-regulated from 3 to 7L stages in susceptible variety. It is the homologous gene of *Arabidopsis* AT1G71830 (Somatic embryogenesis receptor kinase 1, SERK1), The treatment of *Arabidopsis thaliana* with dsRNA can induce SERK1-dependent antiviral resistance [40]. SERK1 can also enhance the resistance of tomato to potato aphids [41].

Ser/Thr PKs are involved in the signal transduction of biological resistance through phosphorylation, including the tomato *Pto* gene, rice *Xa21* gene and wheat *lrk10* gene [42]. The serine/threonine kinase gene Stpk-V confers powdery mildew resistance in wheat [43]. In the RNA-Seq analysis of foxtail millet infected with *S. graminicola* at three different stages, we found that LRR-RLKs and STKs were significantly induced and up-regulated in the infected resistant variety at 3L, while some up-regulated genes appeared relatively late and began to increase at 5L in the susceptible variety. Therefore, these differentially regulated LRR-RLK and STK genes in resistant varieties might play essential roles in foxtail millet resistance against the *S. graminicola* pathogen.

In particular, 22 peroxidases, 19 cytochrome P450s, and 6 laccases were identified as being induced in G1, while most DEGs were inhibited in JG21 after *S. graminicola* induction (Fig. 4). The expression of peroxidase, a well-known pathogen-related protein, also increases when the plant is infected by pathogens [44]. Han et al. analysed the early transcriptome of rice in response to infection with *Ustilaginoidea virens* and found that 16 peroxidase genes were up-regulated in the resistant variety IR28 and down-regulated in the susceptible variety LYP9 [45]. Similar research was reported for the interaction between rice and *Hirschmanniella mucronata* [46], peanut and *Puccinia arachidis* [47]*,* and mulberry and *Meloidogyne enterolobii* based on transcriptome analyses [48]. Cytochrome P450 is a supergene family containing haem monooxygenases involved in many metabolic pathways and defence reactions in plants [49]. P450, as a defence-related gene, was up-regulated in a resistant peanut genotype compared to a susceptible genotype under biotic stress [47]. In addition, recent studies have shown that laccase can enhance disease resistance by increasing defence-induced lignification and lignin components in plant cell walls, which has been reported in studies on cotton verticillium wilt [50], wheat fusarium head blight [51] and soybean *Phytophthora sojae* infection [52].

The plant hormone signal transduction pathway was enriched and showed differential expression following *S. graminicola* infection (Fig. 8). Plant hormones, as signalling molecules, play a key role in regulating the immune response to pathogens [53]. Salicylic acid (SA), ethylene (ET) and jasmonic acid (JA) regulate the basic defence response of plants to deal with various pathogen attacks [54]. However, pathogens can manipulate auxin signals to promote their infection. A high content of auxin can loosen the cell wall, leading to an increase in cell membrane permeability, and the water and nutrients will seep out of cells. In addition, auxin can trigger stomatal opening and promote the spread of pathogens in the host [55]. In this study, six genes (GH3 and SAUR) involved in auxin signalling were enriched in the auxin pathway. These genes were identified to be significantly up-regulated in the resistant variety and weakly or not induced in the susceptible variety at the early stage (3L). GH3 and SAUR are major auxin early response genes in plants. Previous studies have shown that GH3 family genes in rice play a positive regulatory role in *Xoo* and *Xoc* resistance [56]. Chen et al. analysed the transcriptome of highly susceptible and highly resistant varieties infected by citrus canker and found that the expression levels of the auxin metabolic pathway-related genes GH3.1, GH3.6 and GH3.1L in the highly susceptible variety Newhall navel orange were significantly higher than those in the highly resistant variety Siji orange, suggesting that GH3.1, GH3.6 and GH3.1L may be involved in the regulation of resistance/susceptibility to citrus canker [57]. Additionally, PR1 protein is the main component of the plant defence system and has good antifungal activity. PR1 can be induced in many plants infected by many pathogens, which can enhance resistance against pathogens. For quantitative verification, Seita.2G024800 was up-regulated 4.12-fold, 18.64-fold and 6.96-fold from 3 to 7L stage in resistant variety, and the gene was down-regulated in susceptible variety. Seita.2G024900 was also continuously up-regulated by 15.97-fold, 14.72-fold, eightfold in resistant variety, and the gene was down-regulated in susceptible variety (Fig. 9). Moreover, the homologous gene of LOC_OS07g03710 (*OsPR1a*) in rice were up-regulated upon blast fungus and *Xanthomonas oryzae* pv. *oryzae (Xoo)* infection [58, 59]. It speculated that these two genes played important roles in foxtail millet disease resistance against *S. graminicola*.

Notably, starch and sucrose metabolism and fatty acid elongation metabolism were specifically enriched in the susceptible variety JG21 (Fig. 8). Starch accumulation in leaves is a common phenomenon after pathogens infect plant hosts. Excessive starch accumulation will cause chloroplast disintegration, which will lead to leaf yellowing [60]. Foxtail millet leaves also appeared yellow after infection with *S. graminicola*. In this study, the expression of genes related to the starch and sucrose metabolism pathways changed greatly at different stages of infection in the susceptible variety. Most genes began to be induced at 3L. Then, the genes were induced significantly, and the expression reached the highest value at 5L and then gradually decreased at 7L (Fig. 8). In this pathway, glucan endo-1,3-*β*-glucosidase, *β*-glucosidase, endoglucanase, and pectinesterase are cell wall-degrading enzymes (Fig. 8). The cell degradation enzymes secreted by pathogens can decompose the cuticle and cell wall of the host, which is beneficial to the invasion and expansion of pathogens [61]. By transcriptome analysis, cell wall degrading enzyme genes were differentially expressed in resistant and susceptible varieties after *S. graminicola* infection at 3L, 5L and 7L. Glucan endo-1,3-*β*-glucosidase, *β*-glucosidase, endoglucanase and pectinesterase had the highest expression levels at 5L in the susceptible variety (Fig. 8). The enzyme activities of resistant and susceptible varieties infected by *S. graminicola* were also determined. The activities of cellulase and pectinase showed a gradually increasing trend, and most of the enzyme activities reached a maximum at 5L, while the enzyme activities gradually decreased at the later stage (Fig. 2). At the same time, combined with phenotypic symptoms and microscopic observation, it was found that the resistant varieties of foxtail millet had no significant phenotypic change after being infected by *S. graminicola*, but among the susceptible varieties, the leaves of foxtail millet lost their green colour, and yellow and gradually became brown and dry at the later stage, accompanied by a white mildew layer, and the internal cell structure of leaf tissue was destroyed (e.g., chloroplast deformation, starch granules increased, osmiophilic granules gradually increased). Therefore, in the early stage of infection, after the invasion of the pathogen, cell wall thickening and lignification occurred in foxtail millet leaf tissues, and the contents of cellulose and pectinase increased in leaves, thus thickening the host cell wall and resisting pathogen attack. In the later stage of infection, as the metabolism of leaves shifted, the mycelia of the pathogen proliferated in large numbers, which stimulated the activities of related enzymes and made the activity of CWDE increase rapidly (Figs. 1, 2 and 8). At the same time, the cell tissue structure of host leaves was destroyed, which made the cell wall components fully contact enzymes and accelerated degradation and finally led to the occurrence of “grey back” symptoms, with subsequent dryness and death. It is concluded that cell wall-degrading enzymes are involved in the interaction between foxtail millet and *S. graminicola.*

## Conclusions

In this study, comparative transcriptome was conducted to investigate the mechanisms of foxtail millet resistance to *S. graminicola* between resistant G1 and susceptible JG21. We have found some important metabolic pathways involved in disease resistance, including glutathione metabolism, plant hormone signaling, phenylalanine metabolism, etc. At the same time, it was found that leucine-rich protein kinase, Ser/Thr protein kinase, peroxidase, cell wall degrading enzymes were involved in important disease resistance regulation. Three important resistance candidate genes were identified, LRR protein kinase encoded by Seita.8G131800, Ser/Thr protein kinase encoded by Seita.2G024900 and Seita.2G024800. Homologous genes of these genes have been reported to play an important resistance function against pathogen infection in rice and *Arabidopsis thaliana*. the important genes related to the resistance of foxtail millet to *S. graminicola* should be for the major focus of future studies.

## Methods

### Foxtail millet varieties, pathogen and inoculation

Five foxtail millet varieties, JG21, JG40, JG42, FX3 and G1, from BGI Millet Co., Ltd. (Shenzhen, China) were provided by the Institute of Agricultural Bioengineering, Shanxi Agricultural University. We evaluated the resistance of these five varieties in the field and greenhouse in 2018. The evaluation criteria are shown in Table S1. Resistant varieties and highly susceptible varieties were screened for subsequent RNA-Seq experiments. Oospores of the *S. graminicola* were isolated from infected foxtail millet leaves in the field at Shanxi Agricultural University. Leaves with brown lesions were collected, dried naturally, gently crushed, and sifted to obtain oospores, which were stored at 4 °C for later use. The collected oospores were mixed with the seeds of the resistant variety G1 and susceptible variety JG21and sown in pots that were inoculated at 20 ~ 22 °C under 16 h light and 8 h dark conditions in an artificial climate chamber. The resistant variety G1 and the highly susceptible variety JG21 that were not inoculated with oospores were used as the control varieties. Leaf samples of three biological replicates were collected at the 3-, 5-, and 7-leaf stages after inoculation, frozen in liquid nitrogen and stored at －80 °C for later RNA extraction.

### Transmission electron microscopy (TEM)

Ultrastructural changes in resistant and susceptible foxtail millet leaves by *S. graminicola* infection were measured at 3L, 5L and 7L. Samples were dried under vacuum and fixed overnight in 2.5% glutaraldehyde (0.1 mol/L, pH 7.2) at 4 °C and then washed with phosphate buffered saline (PBS) 5 times. Then, the samples were immersed in 1% osmic acid (0.1 mol/L, pH 7.2) for 2 h. Dehydrated tissues in a graded series of methanol (30, 50, 70, 80, 90, 95%) were filtered for 15 min and dehydrated once again with 100% ethanol. The samples were then embedded in epoxy resin and polymerized. Finally, ultrathin sections were obtained using a Reichert Ultracut E ultramicrotome and collected onto 150 mesh formvar-coated copper grids. Sections were incubated for 4 min in uranyl acetate (4% w/v in 50% ethanol) in the dark and stained with uranyl acetate and lead citrate. The samples were examined using a transmission electron microscope.

### Cell wall degrading enzyme activity assay

Seven kinds of cell wall-degrading enzymes, including carboxymethyl cellulose (Cx), *β*-glucosidase (*β*-G), polygalacturonase (PG), pectin methyl galacturonase (PMG), polygalacturonase trans-elimination enzyme (PGTE), *α*-glucosidase and *β*-1,3-glucanase, were investigated in foxtail millet leaves at 3L, 5L and 7L after *S. graminicola* infection. Cell wall degrading enzyme was extracted according to Douaiher [62] and with minor modifications. One gram of fresh leaf tissue was ground with 9 ml of phosphate buffered saline (PBS) on ice, and the pH was adjusted to 7.2, and sample was centrifuged at 4,000 rpm for 20 min. The supernatant fluid was used as a crude enzyme solution and stored at -20 °C for later use. Cellulase (Cx, and *β*-G) and hemicellulase (α-glucosidase and *β*-1,3-glucanase) activities were determined by the method of Hinton [63]. Using the DNS colorimetric method, 3,5-dinitrosalicylic acid was used in a redox reaction with a reducing sugar to produce 3-amino-5-nitrosalicylic acid. The extinction value of the reaction mixture was measured by colorimetry at 540 nm, and the enzyme activity was calculated based on the amount of reducing sugar released by the enzymatic reaction. The pectinase, PG, PMG and PGTE activities were determined by Wang [64]. Pectin methyl trans-eliminating enzyme activity was determined by measuring the absorbance of the reaction mixture using the Hoffman method to calculate pectin methyl trans-eliminating enzyme activity. All experiments were performed in triplicate.

### RNA extraction and Illumina sequencing

Total RNA was extracted from three biological replicates per time point and treatment. A total of 36 RNA samples were extracted (3 harvest stages × 3 replications × 2 treatments × 2 varieties) (Fig. S2). Total RNA was extracted using the RNApure plant kit rapid extraction kit according to the manufacturer’s instructions (CWBIO, Beijing). RNA quality was assessed using an Agilent 2100 Bioanalyzer (Agilent Technologies, Inc., Santa Clara, CA, USA), and two micrograms of total RNA per sample was utilized for TruSeqTM library preparation and RNA sequencing on an Illumina HiSeqTM 2500 platform. The barcoded libraries were multiplexed and sequenced.

### RNA-Seq data analysis

Raw reads were purified by trimming adapters and poly-N and removing low-quality reads to obtain clean reads, which were then mapped to the reference genome (*Setaria italica* v2.2: https://phytozome.jgi.doe.gov/pz/portal.html). The expression level of each gene was normalized to fragments per kilobase per million (FPKM) for comparison between different samples [65]. Differentially expressed genes (DEGs) in foxtail millet varieties were identified by comparing gene expression levels between *S. graminicola-* and mock-inoculated leaves with the criteria of an absolute log2 ratio value ≥ 1 and FDR < 0.01 set as the thresholds for subsequent analysis [66]. DEGs were further annotated by gene Ontology (GO) functional enrichment. GO terms with *P* values of 0.05 were considered significantly enriched. Clusters of orthologous groups (KOG) and pathway analyses were performed using the Kyoto Encyclopedia of Genes and Genomes (KEGG) database (http://www.genome.jp/kegg) [67].

### Real-time quantitative PCR assay

Nine differentially regulated genes identified through RNA-Seq were validated by qRT‒PCR. The primer sets used for qRT‒PCR were designed based on exon sequences of the selected genes using the online program oligo analyser (http://www.idtdna.com), and the specificity of PCR primers was evaluated by blasting primer sequences against the NCBI database (Table S3). Total RNA (1 μg) was used for cDNA synthesis with PrimeScriptTM RT reagent Kit with gDNA Eraser (Takara). PCR was performed in 10 μL of reaction mix containing 0.4 μL cDNA, 10 μL SYBR Premix Ex Taq™ (Takara, Dalian), 5 μL TB Green Mix, 1 μL primer, 3 μL ddH_2_O, and 0.2 μL cDNA using a CFX96™ Real-Time System (BIO-RAD). Three technical replicates for each biological replicate were performed with similar results. Relative gene expression was calculated using the 2-^ΔΔCT^method [68].

## Supplementary Information

**Additional file 1: Fig S1.** Evaluation of five foxtail millet varieties against downy mildew. Disease rate in five foxtail millet varieties after S. graminicola infection. Data are represented as the means ± SEMs.

**Additional file 2: Fig S2.** Diagram showing the study design. The collected oospores were mixed with seeds in the pot were inoculated in an artificial climate chamber. Total RNA was isolated from inoculated seedlings at 3L, 5L and.

**Additional file 3: Table S1.** Evaluation criteria for resistance to downy mildew of foxtail millet.

**Additional file 4: Table S2.** Quality evaluation of sequencing date.

**Additional file 5: Table S3.** Primer sequences designed for quantitative real time RT-PCR.

## Data Availability

All data generated or analysed during this study are included in this published article. The raw sequence data reported in this paper have been deposited in the Genome Sequence Archive (Genomics, Proteomics & Bioinformatics 2021) in the National Genomics Data Center (Nucleic Acids Res 2022), China National Center for Bioinformation /Beijing Institute of Genomics, and Chinese Academy of Sciences. (CRA008114) and are publicly accessible at https://ngdc.cncb.ac.cn/gsa.

## Abbreviations

DEGs: Differentially expressed genes
LRR-PKs: Leucine-rich repeat receptor-like protein kinase
PAL: Phenylalanine ammonia lyase
Cx: Carboxymethyl cellulose
*β*-G: *β*-Glucosidase
PG: Polygalacturonase
PMG: Pectin methyl galacturonase
PGTE: Polygalacturonase trans-elimination enzyme
PBS: Phosphate buffered saline
TEM: Transmission electron microscopy
CWDEs: Cell wall degrading enzymes
GO: Gene Ontology
4CL: 4-Coumarate-CoA
TCM: Trans-cinnamate 4-monooxygenase
NAA: Nicotianamine aminotransferase A
ABA: Abscisic acid
SA: Salicylic acid
JA: Jasmonic acid
IAA: Indoleacetic acid
GA: Gibberellin
FPKM: Fragments per kilobase of exon per million fragments mapped
STK: Serine/threonine-protein kinase
PAMP/MAMP: Pathogen/microbe associated molecular pattern
PRR: Pattern recognition receptors
ETI: Effector triggered immunity
GH3: Auxin-response promoter
SAUR: Sauxin- responsive protein
