# Supplementary figures and images for: Comparative transcriptome profiling of resistant and susceptible foxtail millet responses to Sclerospora graminicola infection
Source: BMC Plant Biol. 2022 Dec 6;22:567. doi: 10.1186/s12870-022-03963-5 (PMC9724433; doi:10.1186/s12870-022-03963-5)

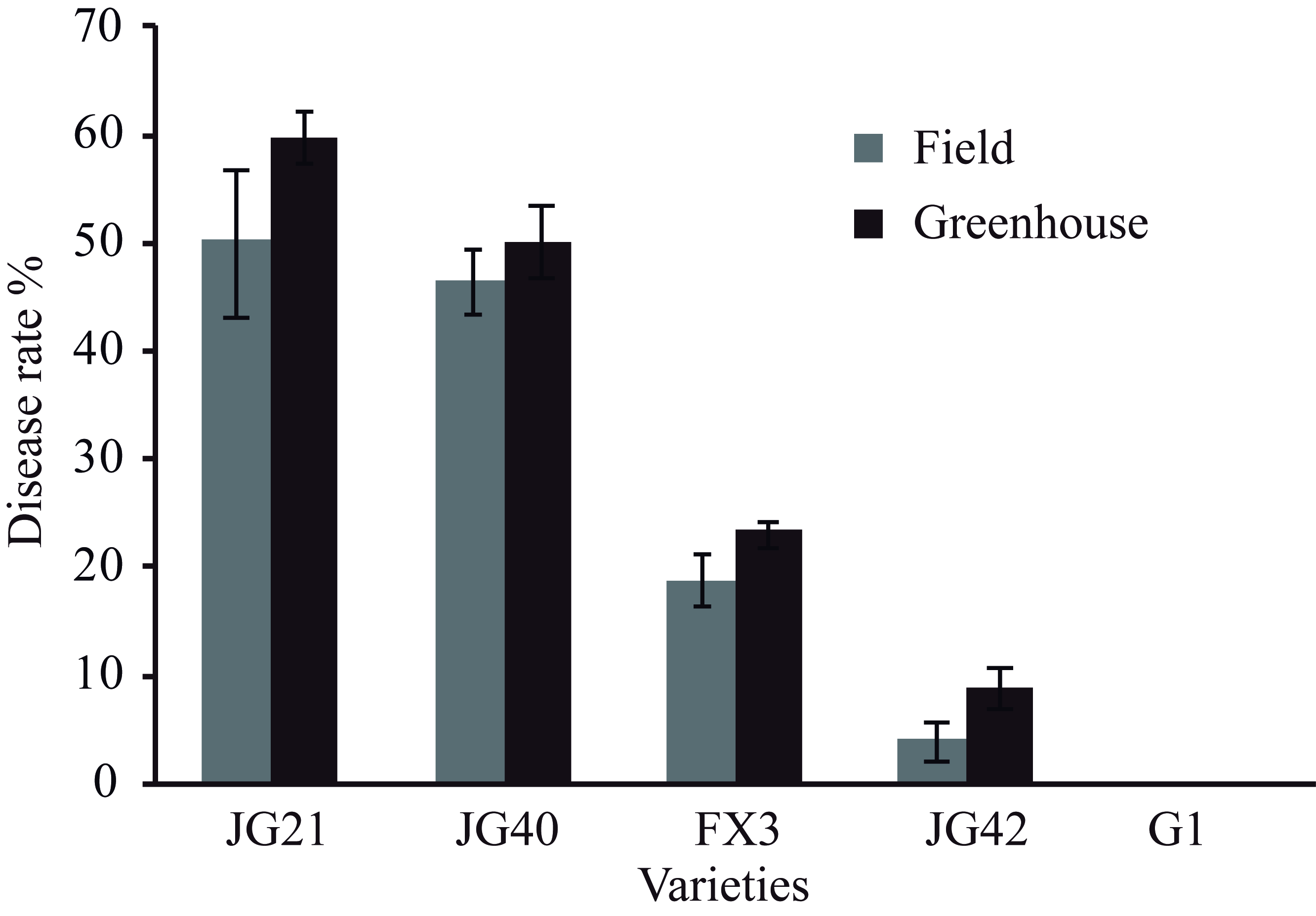

Supplement: Supplementary file 1 — Additional file 1: Fig S1. Evaluation of five foxtail millet varieties against downy mildew. Disease rate in five foxtail millet varieties after S. graminicola infection. Data are represented as the means ± SEMs. [file 12870_2022_3963_MOESM1_ESM.tif]

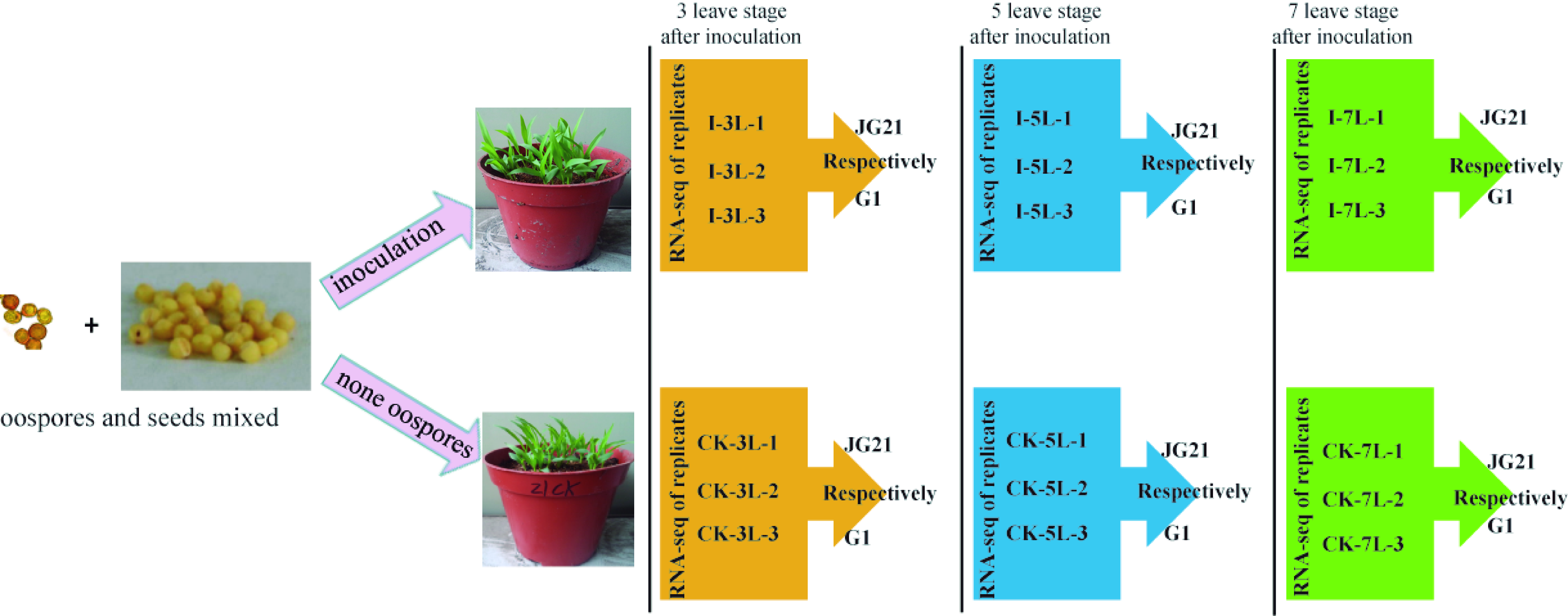

Supplement: Supplementary file 2 — Additional file 2: Fig S2. Diagram showing the study design. The collected oospores were mixed with seeds in the pot were inoculated in an artificial climate chamber. Total RNA was isolated from inoculated seedlings at 3L, 5L and. [file 12870_2022_3963_MOESM2_ESM.tif]
